# Fatty Acid Binding Protein 7 is Involved in the Proliferation of Reactive Astrocytes, but not in Cell Migration and Polarity

**DOI:** 10.1267/ahc.20001

**Published:** 2020-07-04

**Authors:** Tomonori Hara, Banlanjo Abdulaziz Umaru, Kazem Sharifi, Takeo Yoshikawa, Yuji Owada, Yoshiteru Kagawa

**Affiliations:** 1 Department of Organ Anatomy, Tohoku University Graduate School of Medicine, Sendai 980–8575, Japan; 2 Laboratory for Molecular Psychiatry, RIKEN Center for Brain Science, Wako 351–0198, Japan; 3 Department of Medical Biotechnology, School of Advanced Technologies in Medicine, Shahid Beheshti University of Medical Sciences, Tehran, Iran

**Keywords:** fatty acid binding protein 7, reactive astrocyte, proliferation, migration, polarity

## Abstract

Reactive gliosis is a defense mechanism to minimize and repair the initial damage after CNS injuries that is characterized by increases in astrocytic reactivity and proliferation, with enhanced expression of glial fibrillary acidic protein (GFAP) and cellular hypertrophy. Fatty acid binding protein 7 (FABP7) is abundantly expressed in several types of glial cells, such as astrocytes and oligodendrocyte precursor cells, during brain development and FABP7-positive astrocytes have been shown to be significantly increased in the mouse cortex after a stab injury. However, the functional significance of FABP7 in gliosis remains unclear. In the present study, we examined the mechanism of FABP7-mediated regulation of gliosis using an* in vitro* scratch-injury model using primary cultured astrocytes. Western blotting showed that FABP7 expression was increased significantly in scratch wounded astrocytes at the edge of the injury compared with intact astrocytes. Through monitoring the occupancy of the injured area, FAB7-KO astrocytes showed a slower proliferation rate compared with WT astrocytes after 48 hr, which was confirmed by BrdU immunostaining. There were no differences in cell migration and polarity of reactive astrocytes between FABP-KO and WT. Conclusively, our data suggest that FABP7 is important in the proliferation of reactive astrocytes in the context of CNS injury.

## I Introduction

Structurally, astrocytes exhibit a complex spongiform morphology comprised of very fine peripheral processes that come in close contact with synapses and other components of the brain parenchyma [9, 24, 48]. This sophisticated structure of astrocytes is believed to be closely linked to its functions, including the homeostatic regulation of the CNS through the release of neurotrophic factors and cytokines, guidance of neuronal development, as well as contributing towards the metabolism of neurotransmitters and regulation of extracellular pH [45].

Numerous pathological conditions in the CNS resulting from trauma, infection, tumor, seizure and neurodegenerative disease, trigger functional and morphological changes in the astrocytic characteristics giving rise to reactive astrocytes. Astrocyte reactivity is prominently characterized by hypertrophy of the astrocyte cellular processes and high levels of expression of glial fibrillary acidic protein (GFAP), an intermediate filament protein. Additionally, it has been shown that astrocyte reactivity induces a change in astrocyte cell polarity and promotes migration and proliferation through autocrine and paracrine cytokine feedback, culminating in the formation of a scar, traditionally referred to as a “glial scar” [16, 40, 44, 46]. These series of responses compound, leading to a phenomenon known as reactive gliosis, which protects the neuron from acute stress by sequestering pathogenic lesions from non-pathogenic lesions. Conversely, it has been demonstrated that within the post-acute and the early chronic stage after CNS damage, reactive gliosis exhibits inhibitory effects on CNS regeneration [3, 17]. Thus, a deeper understanding of the characteristics of reactive astrocytes and the mechanism underlying reactive gliosis may contribute to the functional prognosis and subsequent treatment of patients with neuropathy.

Polyunsaturated fatty acids (PUFAs) play important roles in cells ranging from maintenance of cellular structure, to metabolism and signaling, and are consequently linked to physiological and pathophysiological functions, including systemic metabolic homeostasis [38], learning/memory [5], immune response [11], and tumor biology [37]. Due to their insolubility in water, PUFAs require a transporter to perform their functional activities within the cell. Fatty acid-binding proteins (FABPs) are capable of solubilizing PUFAs in an aqueous cellular environment, and, as such, act as transporters. FABPs are also vital in maintaining cellular functions, by controlling the uptake, metabolism, and intracellular storage of PUFAs [12, 18, 22]. FABP7, one of the subtypes of FABPs, has been shown to exhibit strong preferential binding affinity to n-3 and n-9 PUFAs, such as docosahexaenoic acid (DHA) and oleic acid respectively [35]. FABP7 is abundantly expressed in several types of glial cells, such as astrocytes and oligodendrocyte progenitor cells, during brain development [41], suggesting that FABP7 plays pivotal roles in the control of the CNS through the regulation of PUFAs actions within the cells.

Previously, we reported that FABP7-positive astrocytes were significantly increased in the cortex after a wound scratch injury and that the number of reactive astrocytes was decreased in the stab-injured cortex of FABP7-KO mice compared with wild-type mice. These results suggest that FABP7 is highly related with astrocyte gliosis. However, the functional significance of FABP7 in gliosis remains unclear. In the present study, we examined the mechanism of FABP7 mediated regulation of gliosis using an *in vitro* scratch-injury model.

## II Materials and Methods

### Reagents

Bromodeoxyuridine (BrdU) and β-D-arabinofuranoside (AraC) were purchased from Merck Millipore (MA, USA). The antibody for rabbit polyclonal anti-mouse FABP7 was established in our laboratory [1]. The antibodies for β-actin and α-tubulin were purchased from Santa Cruz Biotechnology (TX, USA). The antibody for mouse anti-BrdU was purchased from BD Biosciences (CA, USA). The antibodies for rat monoclonal anti-GFAP, mouse IgG-Alexa 488, mouse IgG-Alexa 568, rabbit IgG-Alexa 488, rat IgG-Alexa 488 and DAPI for nuclei staining were purchased from Thermo Fisher Scientific Inc. (MA, USA). The antibody for mouse monoclonal anti-nestin was purchased from Abcam (Cambridge, England). The antibody for pericentrin was purchased from Covance (NJ, USA). The antibodies for horse radish peroxidase (HRP)-conjugated goat-anti rabbit IgG and HRP-conjugated goat anti-mouse IgG were purchased from Merck Millipore.

### Primary astrocyte culture

All the experiments were reviewed by the Ethics Committee for Animal Experimentation of Tohoku University School of Medicine and were performed according to the Guidelines for Animal Experimentation of the Tohoku University School of Medicine (2019MdLMO-154 and 2017MdA-244). Cerebrocortical astrocytes were prepared from C57BL/6 postnatal WT and FABP7-KO (P0-1) mice [26]. Fresh brains were dissected by craniotomy and cerebral cortices were isolated by careful removal of the meninges and hippocampus. Tissues were cut into small pieces and digested in a 0.25% trypsin solution (Thermo Fisher Scientific Inc.) at 37°C for 10 min. After stopping the reaction by Dulbecco’s Modified Eagles’s Medium (Thermo Fisher Scientific Inc.) containing 1% Penicillin-streptomycin (Thermo Fisher Scientific Inc.), 10% fetal bovine serum (Thermo Fisher Scientific, MA, USA) and 0.25 ng/ml Amphotericin B (Sigma-Aldrich Japan, Tokyo, Japan), tissues were dissociated by gentle pipetting and filtered through a 100-μm mesh (BD Falcon, NJ, USA). The cell suspension was centrifuged at 1000 rpm and 20°C for 5 min. The cell pellets were resuspended and seeded onto 75 cm^2^ flasks (BD Falcon, USA) at a density of 3.75 × 10^6^ cells per flask. Flasks were maintained in a 5% CO_2_ incubator at 37°C until confluent. The cells were then shaken on a shaker (BR-40LF; TAITEC, Koshigaya, Japan) at 200 rpm for 1–2 days to remove loosely-attached microglia and oligodendrocyte progenitor cells. The culture was thoroughly washed with DPBS three times and further incubated in new medium for 2 days. Next, 0.05% trypsinized astrocytes were passaged onto 35 mm or 10 cm dishes at a density of 4 × 10^5^ cells/dish or 2 × 10^6^ cells/dish, respectively. The purity of the astrocytes was confirmed to be >95% by GFAP immunocytochemistry.

### *In vitro* reactive gliosis model

We used an *in vitro* model of reactive gliosis as reported previously [16], with a slight modification. Primary astrocytes cultures were prepared until confluent. Then the cells were maintained while gradually decreasing the concentration of serum, as follows (5% for 2 weeks and 1% for 2–3 days before the experiment). WT and FABP7-KO astrocytes were scratched once by using a P200 tip followed by immediate replacement of the medium. The process of wound healing was observed 0 (soon after the scratch), 24, 48 and 72 hr later by phase contrast imaging using a Nikon DS-Fi1-L2 (Nikon, Tokyo, Japan). Wound closure was evaluated by tracing the cell free area at each time point.

### Immunocytochemistry

For immunocytochemistry, the culture dishes containing cells were washed with D-PBS(−) twice and fixed with 4% PFA. Fixed cells were permeabilized with 0.3% (v/v) Triton X-100 in PBS and blocked with appropriate serum in PBS. The reaction with the primary antibodies was performed overnight at 4°C, and the reaction with the secondary antibodies and DAPI was performed for 1 hr at room temperature. The samples were mounted with fluoromount (Diagnostic BioSystems, CA, USA) and then examined by Axioobserver Z1 (Carl Zeiss, Jena, Germany) or Keyence BZ-9000 (Keyence, Osaka, Japan). Images were analyzed by Zen software (Carl Zeiss). Images were taken focusing on the wound closed area and the immuno-positive cells were counted in the wound area following scratch treatment. To evaluate the antibody specificity of FABP7, FABP7-KO astrocytes were used as a negative control as shown in Supplementary Fig. S4A. To evaluate the antibody specificity of pericentrin, immunocytochemistry was performed without each primary antibody as a negative control as shown in Supplementary Fig. S4B.

### Western blotting

Monolayer astrocytes on 10 cm dishes were scratched in a latticed pattern with a side length of 2.5 mm using P200 tips. After immediate replacement of the medium, protein was extracted into sulfate polyacrylamide gel electrophoresis (SDS-PAGE) sample buffer containing a protease inhibitor (Roche, Basel, Switzerland) after 24, 48 and 72 hr. The protein concentration of each sample was determined by the Pierce^TM^ BCA Protein Assay Kit (Thermo Fisher Scientific Inc.). Protein samples were loaded onto 15% SDS polyacrylamide gels and separated by electrophoresis. Proteins were transferred to polyvinylidene difluoride membrane (Merck Millipore). The membrane was blocked with 0.1% (v/v) Tween 20 and 5% (w/v) bovine serum albumin in PBS and incubated with the primary antibody overnight at 4°C followed by incubation with secondary antibody. Detection was performed using the ECL Western Blot Detection Kit (Thermo Fisher Scientific Inc.).

### BrdU uptake assay

To evaluate the proliferation of astrocytes after the wound-scratch assay, the BrdU uptake assay was performed as reported previously [42]. In brief, astrocytes scratched in a latticed pattern with a side length of 2.5 mm were incubated in the presence of 3 μM BrdU for 24, 48 and 72 hr. Non-scratched (intact) astrocytes were similarly prepared as control. Thereafter, cells were fixed with ice-cold 4% (w/v) paraformaldehyde (Nacalai Tesque, Kyoto, Japan) solution for 15 min. To detect the incorporation of BrdU into DNA, they were denatured in 2N HCl for 30 min at room temperature followed by neutralization with 0.1 M sodium borate (pH 8.5) for 10 min twice. Cells were blocked in 5% goat serum/0.1% Triton X/PBS for 20 min at room temperature. Subsequent procedures are same as the protocol for the immunohistochemistry.

### Migration assay

The migration assay was performed as described previously, with a slight modification [39]. WT and FABP7-KO Astrocytes were prepared similarly in 35 mm dishes and injured by a single straightforward scratch. The medium was soon replenished with a new medium containing 8 μM AraC for inhibition of cell proliferation and 3 μM BrdU. The subsequent process of wound closure was recorded by phase contrast imaging 24, 48 and 72 hr later and cell free area was measured at each time point.

### Polarization and protrusion assay

Cellular polarization and protrusion at the beginning of wound closure were evaluated based on Etienne-Manneville and Hall [16]. At the time points of 0, 8, 24 hr later, scratched astrocytes were fixed and immunostained with anti-pericentrin antibody and anti-α-tubulin antibody. The formation of the polarized morphology was assessed by analyzing the reorientation of the microtubule organizing center (MTOC). Astrocytes in which the MTOC was within the quadrant facing the wound were scored as positive. For the evaluation of cellular protrusion, the length of processes located in the wound edge was measured at each time point.

### Statistical analysis

Statistical comparisons were performed using one-way ANOVA followed by Dunnett’s post hoc test or two-way ANOVA followed by Tukey’s post hoc test. P-values less than 0.05 were denoted as being statistically significant. Data are expressed as the means and error bars.

## III Results

### FABP7 is upregulated in scratched astrocytes

**Fig. 1.**
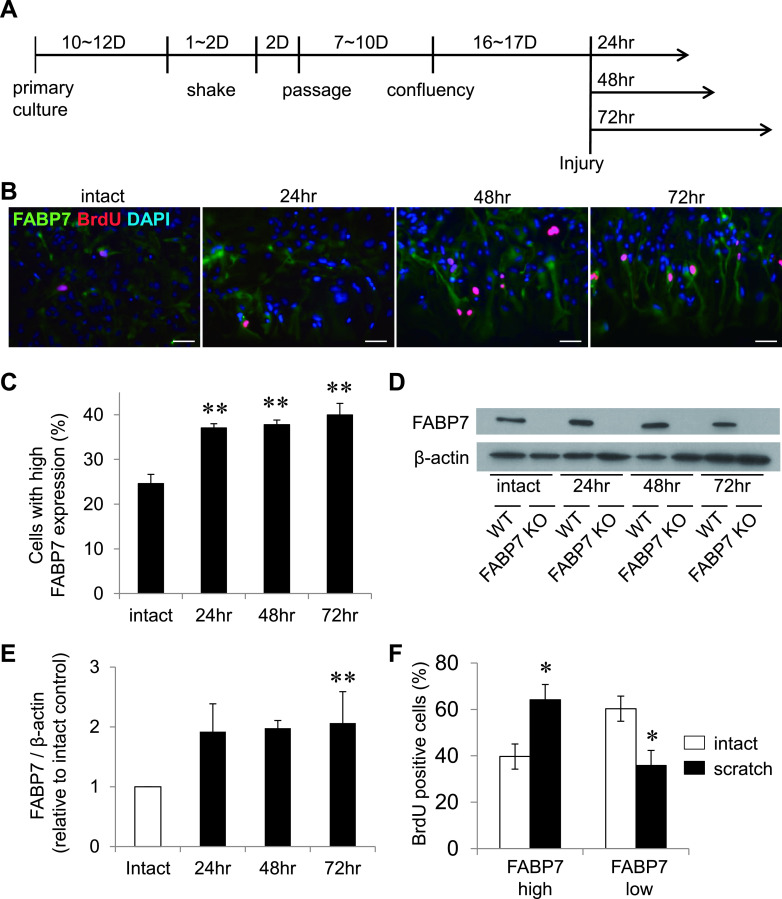
Expression of FABP7 is upregulated in scratched astrocytes. (**A**) Schematic illustration of the time course for the preparation of astrocytes. (**B**) Immunofluorescence staining of FABP7 (green), BrdU (red) and DAPI (blue) in primary cultured astrocytes before/after the scratch treatment. Bar = 50 μm. (**C**) Cells showing high expression of FABP7 before/after the scratch treatment are counted. The number was standardized to the total cell number. (**D, E**) Western blot for FABP7 and β-actin expression in primary cultured astrocytes before/after the scratch treatment. Bar graph (**E**) shows the band density of FABP7 analyzed using NIH-Image J. (**F**) BrdU positive cells were counted in each of the high- and low-FABP7 expressing astrocytes before/after the scratch treatment. Data shown are the means ± s.e.m. and are representative of 3 independent experiments. * P < 0.05, ** P < 0.01.

The *in vitro* scratch assay is often used as a wound healing model [16]. Mature astrocytes (not reactive astrocytes) were obtained according to the procedure shown in Fig. 1A. To confirm the occurrence of the same phenomena observed in the *in vivo* stab-injury model, as we reported previously [41], when using the *in vitro* scratch assay with primary cultured astrocytes, we must first evaluate the changes of FABP7 expression in scratched astrocytes. The immunocytochemistry results showed that astrocytic dendrites gradually occupied the scratched area, and astrocytes at the edge of scratched area expressed higher FABP7 levels compared with intact astrocytes (Fig. 1B, C). Western blotting results also showed that FABP7 was upregulated after scratch (Fig. 1D, E and Supplementary Fig. S5). These results are consistent with our previous *in vivo* experiments in which we demonstrated that FABP7 was upregulated in reactive astrocytes of the stab injury models. Furthermore, as a novel finding, we demonstrated that in the high FABP7 expressing cells, there was a higher number of BrdU positive cells after scratch compared with the intact region (Fig. 1F), suggesting that FABP7 is involved in the proliferation of reactive astrocytes.

### FABP7-KO astrocytes showed lower proliferation after scratch

**Fig. 2.**
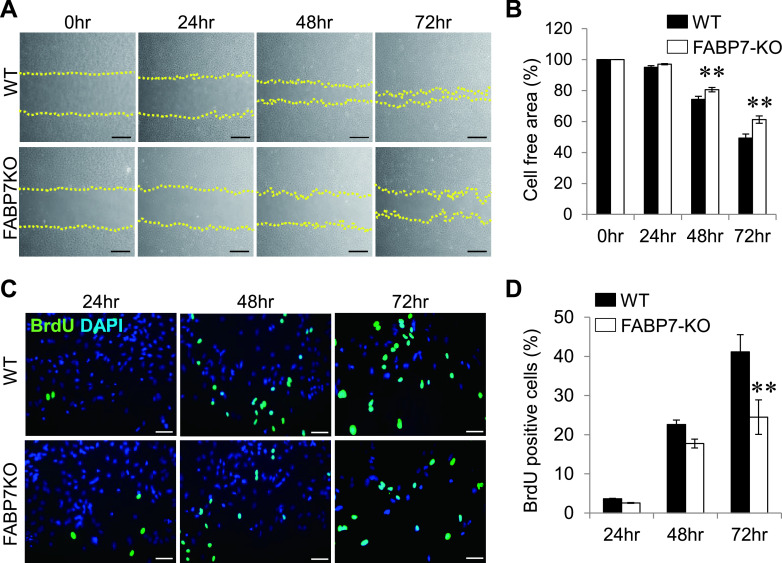
Decrease in proliferation in FABP7-KO astrocytes after scratch. (**A**) Phase-contrast imaging in primary cultured astrocytes indicating the analyzed scratched area at different time points. Bar = 50 μm. (**B**) Analyzed cell-free area after the scratch treatment in primary cultured WT and FABP7-KO astrocytes. (**C**) Immunofluorescence staining of BrdU (green) and DAPI (blue) in primary cultured astrocytes. Bar = 50 μm. (**D**) Counted BrdU positive cells were normalized to the total number of cells. Data shown are the means ± s.e.m. and are representative of 3 independent experiments. ** P < 0.01.

To confirm the regulation of astrocytic proliferation by FABP7, we used primary cultured astrocytes from FABP7-KO mice. Phase-contrast imaging revealed a delayed occupancy of the wound-scratch area in FABP7-KO astrocytes compared with WT astrocytes 48 and 72 hr after the injury (Fig. 2A, B). Even though FABP7-KO astrocytes showed decreased proliferation compared with WT astrocytes without the scratch treatment as shown in Supplementary Fig. S1A, S1B and previous reports [41], the significant difference in the proliferation rate after the scratch wound between WT and FABP7-KO astrocytes was exaggerated compared with the difference observed without the scratch (Fig. 2C, D), suggesting that high expression of FABP7 in reactive astrocytes caused by the wound scratch injury may be the key molecule in cell proliferation.

### FABP7-deficiency is not involved in astrocytic migration

**Fig. 3.**
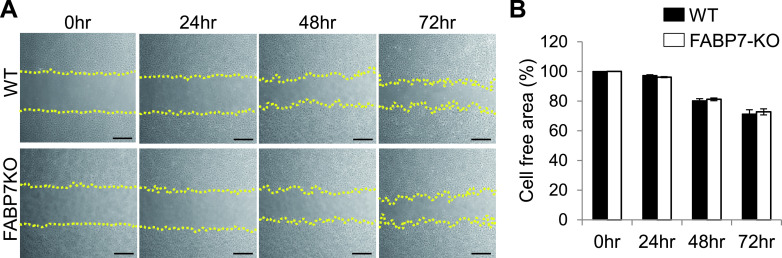
FABP7 deficiency is not involved in astrocytic migration. (**A**) Phase-contrast imaging in primary cultured astrocytes indicating the analyzed scratched area at different time points after treatment with AraC. Bar = 50 μm. (**B**) Analyzed cell-free area after the scratch and AraC treatment in primary cultured WT and FABP7-KO astrocytes. Data shown are the means ± s.e.m. and are representative of 3 independent experiments.

To explain the wound-healing mechanism regulated by FABP7, we next assessed the involvement of cell migration; one of the mechanistic pathways in the formation of the “glial scar”. Several reports have demonstrated their migration assay under treatment with cytosine arabinoside (AraC), a cell proliferation inhibitor. Phase contrast imaging revealed that there is no significant difference in the occupancy of the scratched area between WT and FABP7-KO astrocytes under AraC treatment (Fig. 3A, B), suggesting that FABP7 is not involved in the migration of reactive astrocytes.

### FABP7-deficiency is not involved in astrocytic polarity

**Fig. 4.**
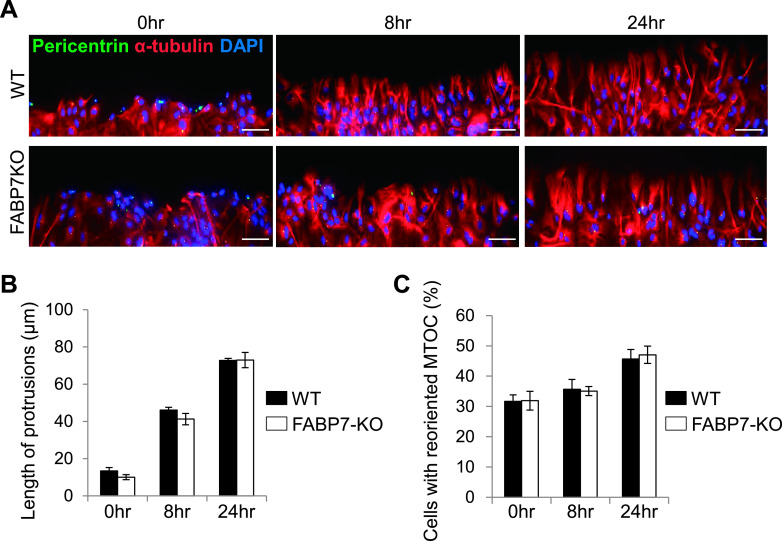
FABP7 deficiency is not involved in astrocytic polarity. (**A**) Immunofluorescence staining of pericentrin (green), α-tubulin (red) and DAPI (blue) in primary cultured astrocytes after the scratch treatment. Bar = 50 μm. (**B**) The length of the processes located at the wound edge were analyzed after the scratch treatment at each time point. (**C**) Counted cells with reoriented MTOC. Astrocytes in which the MTOC was within the quadrant facing the scratched area were scored positive. Data shown are the means ± s.e.m. and are representative of 3 independent experiments.

Because astrocytes at the wound area relocate their microtubule organizing center (MTOC), followed by the protrusion process, we next assessed whether FABP7 is involved in the location of MTOC and the extension of the protrusions. Astrocytes that contained a pericentrin-immunostained-dot within the quadrant facing the wound area were scored as positive (Supplementary Fig. S2, right) and the length of the processes located towards the wound edge was measured for the evaluation of cellular protrusion (Supplementary Fig. S2, left). We found no difference in the location of MTOC (Fig. 4A, B) and the extension of the protrusion (Fig. 4A, C) at any time points.

## IV Discussion

There is a growing body of evidence pointing towards the positive consequences of reactive gliosis. Previous experimental studies have demonstrated the attenuation of acute neurodegeneration and repair of the blood-brain barrier after CNS injury through the elimination of proliferating astrocytes by the GFAP-promoter driven herpes simplex virus thymidine kinase [8]. In addition, genetic ablation of astrocytic intermediate filament, such as GFAP and vimentin, in mice results in decreased resistance of the CNS to severe mechanical stress [32]. In contrast, a number of experimental models have shown the inhibitory effects of reactive gliosis on CNS regeneration [2, 19]. For example, inhibition of chondroitin sulphate proteoglycans or release of ephrin A4 by reactive astrocytes has been shown to improve post-traumatic axonal regeneration after spinal cord injury [6, 19]. Given these contrasting findings, understanding the crucial function of reactive gliosis is therefore beneficial for CNS regeneration. FABP7 is abundantly expressed in neural stem cells, astrocytes and oligodendrocyte precursor cells under normal physiological conditions [36, 41] and is considered as the key player regulating astrocytic activity because it is associated with a number of brain diseases including functional psychosis, such as schizophrenia and autism [25, 34, 47]. In this study, we demonstrated that FABP7 is involved in the proliferation of reactive astrocytes using an *in vitro* wound-healing model, but not associated with migration and cell polarity of reactive astrocytes.

Several previous papers have reported a decrease in the number of GFAP-positive cells and total BrdU uptake in the hippocampal dentate gyrus of FABP7-KO mice at postnatal 4 weeks [47]. Decreased proliferation and late confluency has also been shown in cultured primary FABP7-KO astrocytes [41]. Under pathophysiological condition, FABP7 has been shown to be strongly expressed in several malignant tumors, such as glioma [13, 28], breast cancer [4], and malignant melanoma [14, 21] and the expression levels correlate with the rate of tumor proliferation, poor patient prognosis and survival. In this study, the number of BrdU-positive reactive astrocytes from scratch treatment was higher than in intact astrocytes, and reactive astrocytes with high FABP7 expression from the scratch treatments exhibit more positive BrdU than low FABP7 expressing reactive astrocytes (Fig. 1F), suggesting that the activity of reactive astrocytes correlated with the expression level of FABP7. Simultaneously, we performed immunocytochemistry for nestin and GFAP to evaluate the levels of undifferentiation of astrocytes as reported previously [7, 10, 15, 29], and found that FABP7-KO astrocytes showed a higher number of nestin-positive cells compared with WT in intact condition (Supplementary Fig. S3A, B). There was no change in the positivity after the scratch treatment although there was a gradual increase in nestin-positive cells in WT after scratch and a significant difference was obtained 48 h after the scratch treatment (Supplementary Fig. S3A, B) as reported previously [49]. Therefore, such alteration in phenotypic diversity of FABP7-KO astrocytes indicated by the different nestin expression may partially contribute to the difference in response to scratch injury. This hypothesis should be further examined using WT and FABP7-KO astrocytes with clearly evaluated nestin-expression.

We previously reported that FABP7 controls the function of astrocytic caveolae, one of the membrane microdomains that are a main source of cellular activity in response to external stimuli, through the regulation of caveolin-1 expression [26, 27]. This finding revealed that TNF-α production, cell signaling activity including pERK, p38MAPK, pJNK and phosphorylation of IκB, and the recruitment of TLR4 (toll-like receptor 4) into the caveolae fraction in FABP7-KO astrocytes in response to LPS stimuli was decreased compared with WT astrocytes, suggesting that FABP7-KO astrocytes have a dull response to external stimuli, followed by decreased cell activity. Moreover, previous studies have shown that several cytokines and growth factors such as FGF-2, EGF, CNTF and VEGFα, are involved in the proliferation of reactive astrocytes [20, 30, 31, 33]. Taken together, FABP7-KO astrocytes may show a decreased response to these humoral factors as a consequence of the scratch treatment, and decreased cell activity may lead to a decrease in proliferation.

Astrocytes respond to CNS injury insults with a variety of different signaling events, including proliferation and alterations in various molecular expression, cellular hypertrophy, and migration [43]. In this study, we evaluated whether FABP7 is involved in the migration of reactive astrocytes followed by cell polarity, but no significant difference was detected between WT and FABP7-KO astrocytes. Even though contrasting results were observed between proliferation and migration, previous reports have demonstrated that elimination of STAT3 activation attenuates the migration of reactive astrocytes, but not proliferation [23]. Our previous report also showed a decrease in ERK phosphorylation in the FABP7-KO hypothalamus after leptin treatment, but with no significant difference in phosphorylation of STAT3 between WT and FABP7-KO, although not performed on reactive astrocytes [50]. These results indicate that FABP7 is not involved in the migration of reactive astrocytes because FABP7 does not regulate the STAT3 signaling and that FABP7 strongly regulates ERK signaling possibly through caveolae, as mentioned above. Considering the possible association between FABP7-mediated lipid raft function and the proliferation of reactive astrocytes, further studies elucidating the underlying mechanism may provide an understanding of novel functions of FABP7 and lead towards therapeutic approaches for several FABP7-associated CNS diseases.

## V Conflicts of Interest

The authors declare no competing interests.

## VI Acknowledgments

We thank Dr. Majid Ebrahimi, Dr. Hirofumi Miyazaki and Dr. Yui Yamamoto for technical support and the Biomedical Research Unit of Tohoku University Hospital for their support. This work was supported in part by Japan Society for the Promotion of Science (JSPS) KAKENHI Grant (No. 17K15539 to Y.K., and No. 19H04026 to Y.O.), in part by AMED under Grant Number JP17dm0107071 (to K.F. and Y.O.), and in part by the GSK Japan Research Grant 2015 (to Y.K.).

## Supplementary Materials

Supplementary Fig. S1. FABP7 is involved in astrocyte proliferation even without scratch. (**A**) Immunofluorescence staining of BrdU (green) and DAPI (blue) in primary cultured astrocytes. Bar = 50 μm (**B**) Counted BrdU positive cells were standardized to the total number of cells. (Data shown are the means ± s.e.m. and representative of 3 independent experiments. * P < 0.05)

Supplementary Fig. S2. Evaluation of the length of the process and MTOC. Immunofluorescence staining of pericentrin (green), α-tubulin (red) and DAPI (blue) in primary cultured astrocytes after the scratch treatment. Location of the pericentrin immunostained-dot, as shown in the right, is indicated with respect to the orientation from the scratched area for scoring. The arrow in the left cell indicates the length of the process to the edge of scratched area.

Supplementary Fig. S3. The number of nestin-positive cells is higher in FABP7-KO astrocytes compared with WT astrocytes. (**A**) Immunofluorescence staining of nestin (green), GFAP (red) and DAPI (blue) in primary cultured astrocytes. Bar = 50 μm (**B**) The counted nestin-positive cells were standardized to the DAPI-positive cells. (Data shown are the means ± s.e.m. and are representative of 3 independent experiments. ** P < 0.01, * P < 0.05)

Supplementary Fig. S4. Evaluation of the specificity of the antibodies. (**A**) The antibody specificity of the FABP7 antibody was evaluated with WT and FABP7-KO astrocytes. FABP7 (green), DAPI (blue). Bar = 50 μm (**B**) The antibody specificity of pericentrin was evaluated by primary antibody omission using WT astrocytes. Pericentrin (green) DAPI (blue). Bar = 50 μm

Supplementary Fig. S5. Whole film image of Western blott before cropping. (**A, B**) The film image for FABP7 (**A**) and β-actin (**B**).
