# Supplementary material for: Fatty Acid Binding Protein 7 is Involved in the Proliferation of Reactive Astrocytes, but not in Cell Migration and Polarity
Source: Acta Histochem Cytochem. 2020 Jul 4;53(4):73–81. doi: 10.1267/ahc.20001 (PMC7450179; doi:10.1267/ahc.20001)
Supplement: Supplementary Fig. S1. — FABP7 is involved in astrocyte proliferation even without scratch. (A) Immunofluorescence staining of BrdU (green) and DAPI (blue) in primary cultured astrocytes. Bar = 50 μm (B) Counted BrdU positive cells were standardized to the total number of cells. (Data shown are the means ± s.e.m. and representative of 3 independent experiments. * P < 0.05) [file AHC20001_S1.pdf]

Acta Histochem. Cytochem.
doi: 10.1267/ahc.20001
© 2020 The Japan Society of Histochemistry and Cytochemistry

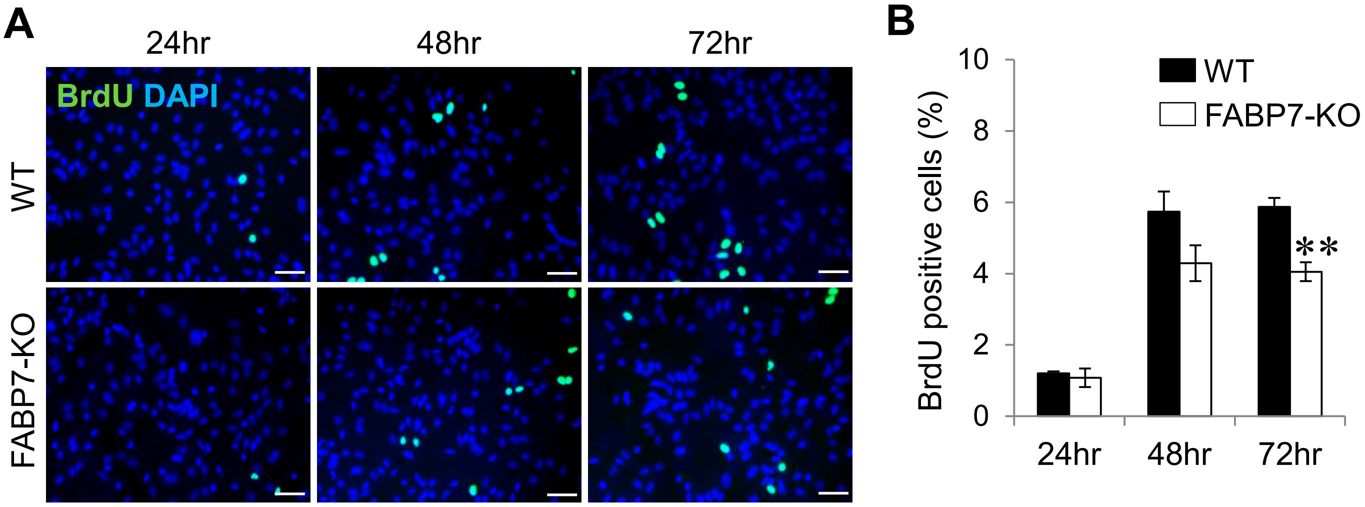

Supplementary Fig. S1. FABP7 is involved in astrocyte proliferation even without scratch. (**A**) Immunofluorescence staining of BrdU (green) and DAPI (blue) in primary cultured astrocytes. Bar = 50 µm. (**B**) Counted BrdU positive cells were standardized to the total number of cells. Data shown are the means ± s.e.m. and representative of 3 independent experiments. * $P < 0.05$.
