# Supplementary material for: Fatty Acid Binding Protein 7 is Involved in the Proliferation of Reactive Astrocytes, but not in Cell Migration and Polarity
Source: Acta Histochem Cytochem. 2020 Jul 4;53(4):73–81. doi: 10.1267/ahc.20001 (PMC7450179; doi:10.1267/ahc.20001)
Supplement: Supplementary Fig. S2. — Evaluation of the length of the process and MTOC. Immunofluorescence staining of pericentrin (green), α-tubulin (red) and DAPI (blue) in primary cultured astrocytes after the scratch treatment. Location of the pericentrin immunostained-dot, as shown in the right, is indicated with respect to the orientation from the scratched area for scoring. The arrow in the left cell indicates the length of the process to the edge of scratched area. [file AHC20001_S2.pdf]

Acta Histochem. Cytochem.
doi: 10.1267/ahc.20001
© 2020 The Japan Society of Histochemistry and Cytochemistry

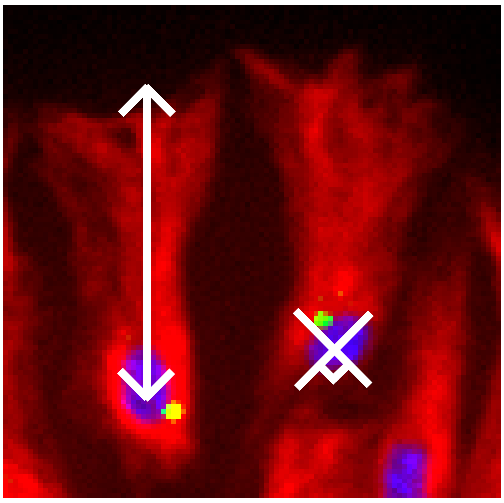

Supplementary Fig. S2. Evaluation of the length of the process and MTOC. Immunofluorescence staining of pericentrin (green), α-tubulin (red) and DAPI (blue) in primary cultured astrocytes after the scratch treatment. Location of the pericentrin immunostained-dot, as shown in the right, is indicated with respect to the orientation from the scratched area for scoring. The arrow in the left cell indicates the length of the process to the edge of scratched area.
