# Supplementary material for: Fatty Acid Binding Protein 7 is Involved in the Proliferation of Reactive Astrocytes, but not in Cell Migration and Polarity
Source: Acta Histochem Cytochem. 2020 Jul 4;53(4):73–81. doi: 10.1267/ahc.20001 (PMC7450179; doi:10.1267/ahc.20001)
Supplement: Supplementary Fig. S3. — The number of nestin-positive cells is higher in FABP7-KO astrocytes compared with WT astrocytes. (A) Immunofluorescence staining of nestin (green), GFAP (red) and DAPI (blue) in primary cultured astrocytes. Bar = 50 μm (B) The counted nestin-positive cells were standardized to the DAPI-positive cells. (Data shown are the means ± s.e.m. and are representative of 3 independent experiments. ** P < 0.01, * P < 0.05) [file AHC20001_S3.pdf]

Acta Histochem. Cytochem.
doi: 10.1267/ahc.20001
© 2020 The Japan Society of Histochemistry and Cytochemistry

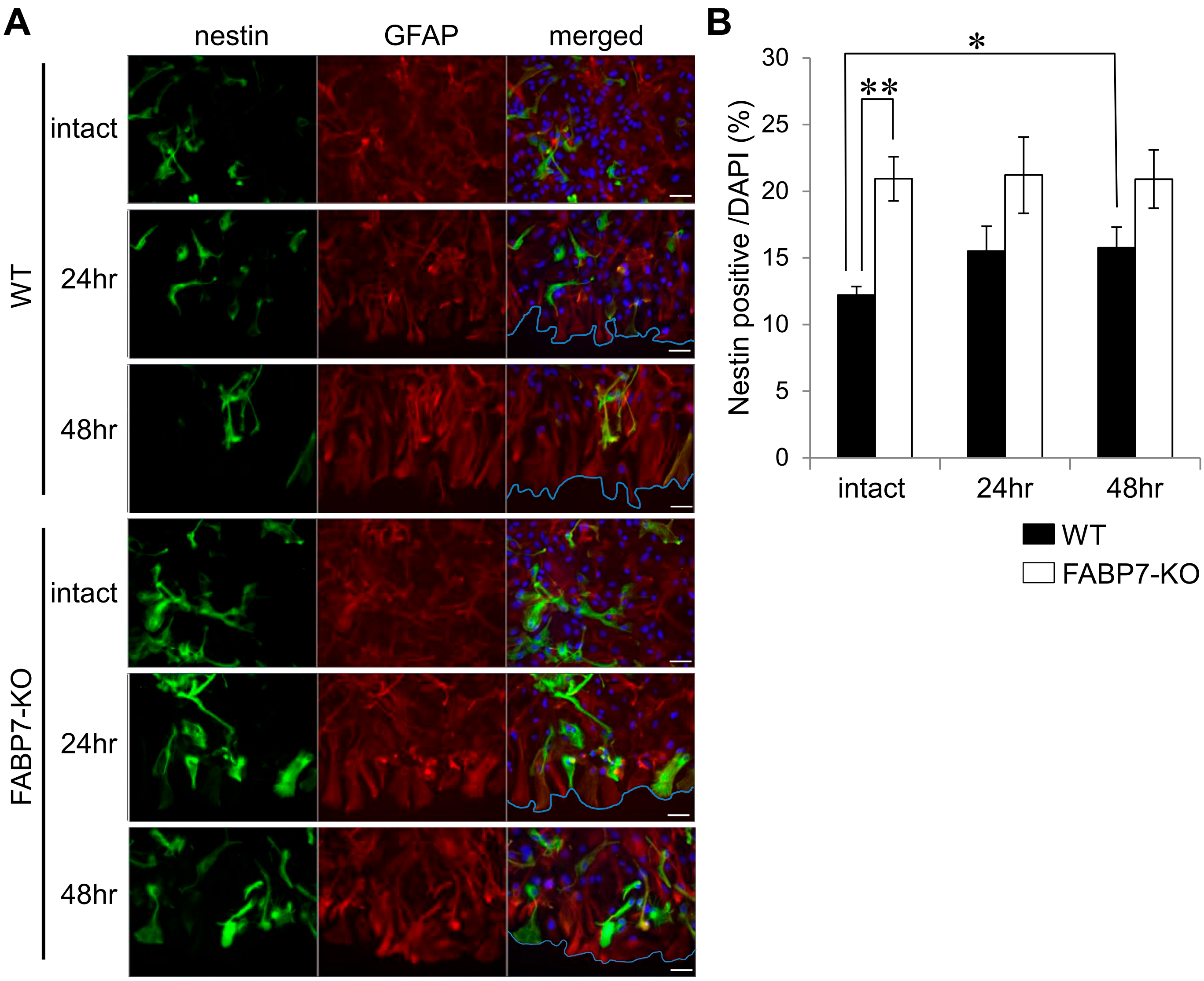

Supplementary Fig. S3. The number of nestin-positive cells is higher in FABP7-KO astrocytes compared with WT astrocytes. (**A**) Immunofluorescence staining of nestin (green), GFAP (red) and DAPI (blue) in primary cultured astrocytes. Bar = 50 µm. (**B**) The counted nestin-positive cells were standardized to the DAPI-positive cells. Data shown are the means ± s.e.m. and are representative of 3 independent experiments. ** $P < 0.01$, * $P < 0.05$.
