# Supplementary material for: Fatty Acid Binding Protein 7 is Involved in the Proliferation of Reactive Astrocytes, but not in Cell Migration and Polarity
Source: Acta Histochem Cytochem. 2020 Jul 4;53(4):73–81. doi: 10.1267/ahc.20001 (PMC7450179; doi:10.1267/ahc.20001)
Supplement: Supplementary Fig. S4. — Evaluation of the specificity of the antibodies. (A) The antibody specificity of the FABP7 antibody was evaluated with WT and FABP7-KO astrocytes. FABP7 (green), DAPI (blue). Bar = 50 μm (B) The antibody specificity of pericentrin was evaluated by primary antibody omission using WT astrocytes. Pericentrin (green) DAPI (blue). Bar = 50 μm [file AHC20001_S4.pdf]

Acta Histochem. Cytochem.
doi: 10.1267/ahc.20001
© 2020 The Japan Society of Histochemistry and Cytochemistry

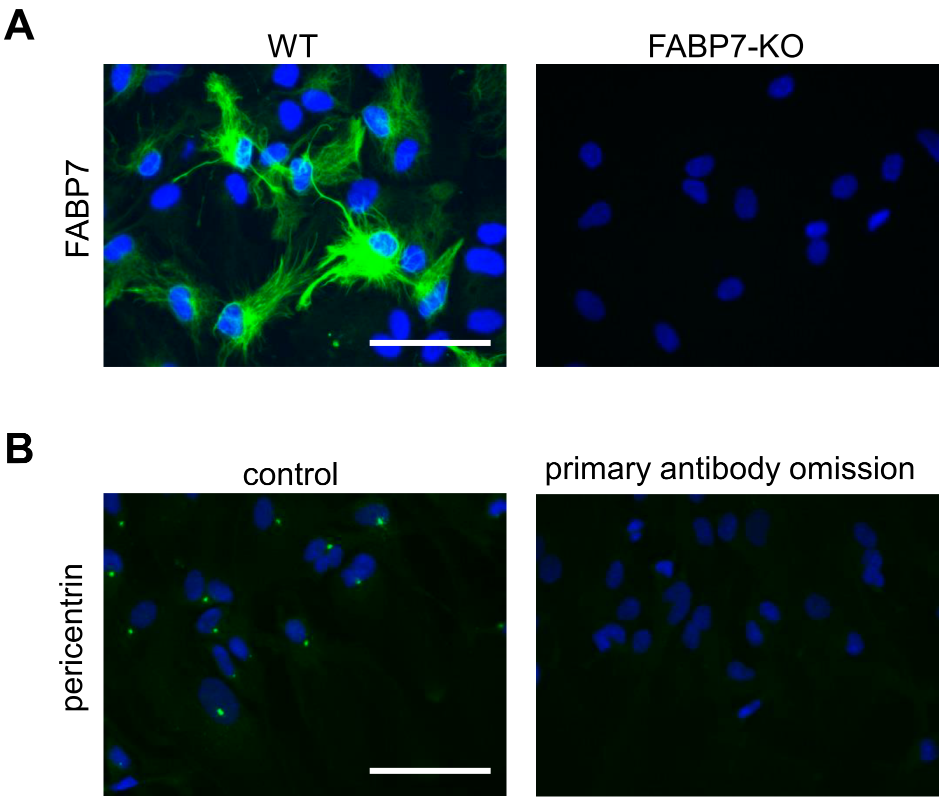

Supplementary Fig. S4. Evaluation of the specificity of the antibodies. (**A**) The antibody specificity of the FABP7 antibody was evaluated with WT and FABP7-KO astrocytes. FABP7 (green), DAPI (blue). Bar = 50 µm. (**B**) The antibody specificity of pericentrin was evaluated by primary antibody omission using WT astrocytes. Pericentrin (green) DAPI (blue). Bar = 50 µm.
