# Supplementary material for: Fatty Acid Binding Protein 7 is Involved in the Proliferation of Reactive Astrocytes, but not in Cell Migration and Polarity
Source: Acta Histochem Cytochem. 2020 Jul 4;53(4):73–81. doi: 10.1267/ahc.20001 (PMC7450179; doi:10.1267/ahc.20001)
Supplement: Supplementary Fig. S5. — Whole film image of Western blott before cropping. (A, B) The film image for FABP7 (A) and β-actin (B). [file AHC20001_S5.pdf]

Acta Histochem. Cytochem.
doi: 10.1267/ahc.20001
© 2020 The Japan Society of Histochemistry and Cytochemistry

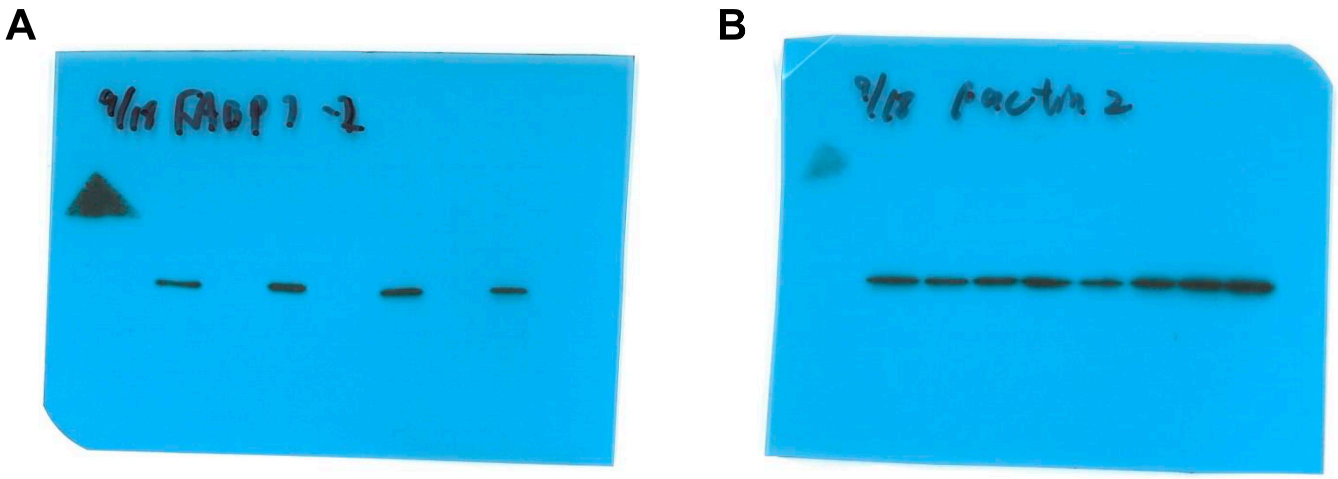

Supplementary Fig. S5. Whole film image of Western blott before cropping. (**A, B**) The film image for FABP7 (**A**) and β-actin (**B**).
